# The Suppressibility of Otoacoustic Emissions and Loudness by Low-Frequency Biasing Tones as a Function of Probe Level

**DOI:** 10.1007/s10162-026-01029-z

**Published:** 2026-01-26

**Authors:** Torsten Marquardt, Juan Vizuete, Markus Drexl, Carlos Jurado

**Affiliations:** 1 UCL Ear Institute, London, WC1X8EE UK; 2 Escuela Técnica Superior de Ingeniería en Telecomunicación, Universidad de Málaga, Málaga, 29071 Spain; 3 Audiology Group, Norwegian University of Science and Technology, Trondheim, 7491 Norway

**Keywords:** Biasing, Cochlear compression, Otoacoustic emissions, Loudness

## Abstract

**Purpose:**

Biasing of the cochlear partition by tones below 100 Hz is commonly used to investigate cochlear non-linearity. Their long periodicity allows resolution of the suppression of cochlear responses as the partition is displaced periodically away from its resting position. The purpose of this study was to quantify by how much the biasing tone (BT) level needed to be increased to keep an equal suppression depth in OAE and an equal criterion suppression of loudness while the levels of the response-evoking stimuli were increased.

**Method:**

Suppression-period patterns were obtained for the distortion-product and stimulus-frequency otoacoustic emissions (DPOAE: *N* = 8 ears, SFOAE: *N* = 6 ears) using a 55-Hz BT, conditions in which the primary frequencies were at least 3 octaves above the BT frequency. A prior search for primary parameters that allowed the highest recording SNR was conducted, including an optimization of the lower primary tone level (L1) for the DPOAE. The BT level was adjusted so that the maximum suppression depth was kept constant at 9 dB for both OAEs, for various primary levels. Also, the BT level required to reach an equal criterion suppression in the loudness of 1- and 2-kHz tone-pip probes was measured psychoacoustically (*N* = 4) at various probe levels using the same BT.

**Results:**

The increase in suppressor level per increase in probe level was similar (~ 0.4 dB/dB) for equal-loudness suppression of the tone pips, 9-dB suppression of the SFOAE, and the 2F1–F2 DPOAE, when the increase in L1 was considered. Average BT levels increased linearly over the whole range of probe levels tested.

**Conclusion:**

As the observed iso-suppression rate is broadly consistent with the growth of basilar membrane vibration with increasing stimulus level in the active region of the traveling wave, we conclude that equal suppression of cochlear responses during partition biasing occurs when bias displacement grows in proportion to the probe’s traveling wave amplitude in its active region.

## Introduction

Biasing tones (BT) are commonly used in auditory research to study cochlear nonlinearity and amplification. Their frequencies are usually less than 100 Hz so as to produce, without significant phase differences over a large longitudinal extent of the cochlear partition, a slow periodic displacement bias toward the scala vestibuli and the scala tympani, which is thought to change the operating point of the outer-hair cell (OHC) mechanoelectrical transduction (MET) channels in the organ of Corti. The operating point change is the consequence of deflection of the OHC’s stereocilia by the associated shear between the reticular lamina (RL) and the tectorial membrane (TM), and can lead to suppression of responses evoked by higher-frequency sounds, which are typically at least ten times higher in frequency than the BT (i.e., > 3 octaves), so that the bias can be considered quasi-static. The periodic modulation (mostly a periodic suppression) of responses to higher-frequency probe stimuli has been observed in basilar membrane (BM) vibrations (e.g., [1–5]), otoacoustic emissions (OAEs; e.g., [6–11]), hair-cell potentials (e.g., [3, 12, 13]) and neuronal responses of the auditory nerve and brainstem (e.g., [15, 16]). Changes in the behavioral hearing threshold of tone pips as a function of their position within the BT cycle (so-called masking-period patterns) have been extensively studied by Zwicker [6, 17, 18]. Also, a suppression of loudness, or, with higher BT frequencies, a roughness, can be perceived [19, 20].

As we have, these researchers have probably experienced that higher probe stimulus levels require higher BT levels to maintain equal suppression of the physiological response to the probe or its sensation. Thus, choosing the right probe levels for these experiments is a compromise between obtaining a sufficient probe-to-noise level for fast data collection and a low BT level for subject safety. Therefore, we set out to quantify by how much the BT level needs to be raised with increasing probe level because this dependence has been systematically assessed only under conditions where the suppressor frequency was less than 2 octaves below the primary frequencies (see Discussion). We measured iso-suppression curves as a function of probe level for distortion-product and stimulus-frequency otoacoustic emissions (DPOAE and SFOAE), as well as the loudness suppression by a 55-Hz BT. Comparison with reported slopes of the compressive growth of BM responses lets us suggest that equal suppression of cochlear responses during partition biasing occurs when bias displacement grows in proportion to the probe’s traveling wave (TW) amplitude in its active region.

## Methods

### Subjects

For the OAE experiments, 16 subjects, aged 22–31 years and not reporting any hearing disorders, were recruited. Six subjects were excluded because their DPOAE levels did not meet the 30-dB SNR criterion at low primary tone levels. One subject was measured in both ears, and so optimal L1-levels could be obtained for eleven ears. Subsequently, three further subjects were excluded because a 9-dB suppression of their DPOAEs could not be achieved for more than 20% of primary parameter settings within our safety level limit for the 55-Hz BT (max. 115 dB SPL, corresponding to ~ 105 phon). From the remaining eight ears measured with DPOAE suppression, six were available for comparison with SFOAE suppression, as two ears did not produce SFOAE with an SNR of 30 dB at the lowest primary tone level. DPOAE and SFOAE measurements were carried out on different days.

Four normal-hearing subjects aged 24–55 years took part in the loudness experiment after three participants were excluded due to inconsistent responses during training (see exclusion criterion in the “Loudness suppression” section). None of them participated in the preceding OAE experiment.

All subjects provided their written informed consent to participate in these experiments, which were approved by the UCL ethics committee (ID 0565–004). No sex- and gender-based analyses (SGBA) were performed.

### Apparatus and Calibration

Signals were generated, recorded, and analyzed using custom-written MATLAB scripts, and D/A and A/D were converted at 48 kHz by a Fireface UC external sound interface (24-bit; RME Audio AG, Germany). To elicit and record OAEs, an ER-10C probe (Etymotic Research Inc., Elk Grove Village, IL) was used. The BT was produced by a DT-48 earphone (Beyerdynamic GmbH & Co. KG, Heilbronn, Germany) that was coupled to the ear canal via a flexible 200-mm-long tube (0.5 mm i.d.) that protruded through the well-sealing ER10C-14A earplug into the ear canal. The DT-48 was directly driven by the headphone amplifier of the sound interface.

The probe’s microphone was calibrated using an ear simulator (type 4157; Brüel & Kjær A/S, Denmark). In situ calibrations after each probe placement ensured the defined sound pressures at the probe’s microphone in individual ear canals. Note that this in situ calibration will not lead to accurate absolute levels at the ear drum above ~ 2 kHz because of standing-wave resonances in the ear canal. It is, however, not crucial for the slope estimation of iso-suppression curves at each single probe frequency. Detailed method descriptions can be found in [20]. The same hardware was used in OAE and loudness experiments. The probe stimulus for the loudness suppression experiments was produced by one of the two ER-10C receivers. All tests took place in triple-walled soundproof booths at the UCL Ear Institute.

### DPOAE Suppression

The measurement of DPOAE iso-suppression curves is described in detail in the works of Marquardt et al. [9] and Jurado et al. [20]. Briefly, the experiment began with a quick search for F2 primary frequencies that gave strong OAE within the range of 0.5–8 kHz (1/4-octave steps, F2/F1 = 1.2, L1/L2 = 55/40 dB SPL). As the extraction of the suppression pattern requires a large signal-to-noise ratio (SNR), the 2F1–F2 component had to have a minimum of 30-dB SNR. (Note that this coarse search did not necessarily lead to the choice of a local maximum in the spectral OAE fine structure; it was only intended to achieve this SNR.) Two F2 frequencies were chosen that were at least 1 octave apart. Because the BT level required to achieve constant maximum OAE suppression depth increases with primary frequency (~ 9 dB/octave for DPOAE; see Jurado et al. [20]), preference was given to lower primary frequencies to avoid excessive BT levels.

Once two suitable F2 primary frequencies were identified, the L1 levels were optimized in 3-dB steps to produce the maximum level of the 2F1–F2 DPOAE. This was done for each L2 tone level to be tested with biasing. The stimulus duration for all these primary parameter optimizations was 4.4 s.

Measurements of DPOAE iso-suppression curves were carried out with 20-s-long stimuli. For each L2 tested, the unsuppressed 2F1–F2 level was first reassessed and served as the reference for the subsequent suppression adjustment procedure. At the beginning, the 55-Hz BT was set at a level below that expected to cause a large suppression. The BT level was then iteratively adjusted by the experimenter until the difference between the unsuppressed reference DPOAE and the lowest DPOAE level within the suppression pattern reached our suppression criterion of 9 dB. Typically, three to four attempts were required to obtain suppression patterns of slightly more and slightly less than the suppression criterion (i.e., within 8–10 dB suppression), from which the required BT level for 9-dB suppression was derived by interpolation. Iso-suppression curves were obtained sequentially for increasing L2 (30–65 dB SPL, varied in 5-dB steps).

To obtain average spectra, the recording was sectioned into 400-ms-long snippets, with the first snippet discarded to avoid latency and onset effects. The spectra of the averaged snippets had sharp spectral lines with 2.5 Hz spacing, without spectral splatter. Suppression period patterns (i.e., the DPOAE amplitude as a function of BT phase) were obtained by inverse Discrete Fourier Transform (DFT) of the 2F1–F2 “carrier” and the two closest modulation side-line pairs (see Marquardt et al. [9]). As in all biasing experiments, the suppression pattern shows the sum of the wave-fixed and the place-fixed DPOAE components [21].

The spectrum of the averaged snippets, as well as the suppression period pattern, was displayed immediately after each recording. Because the experimenter was inside the soundproof booth, the subject could also see the display, getting immediate feedback about noise (e.g., caused by movement or loud breathing) and staying motivated by following the progress of the BT-level adjustment. As subjects had no control over the suppressor level, it was furthermore important that the experimenter had direct verbal feedback as to whether the subject was still comfortable with it.

### SFOAE Suppression

SFOAEs were obtained with the two-tone suppression method [22, 23], using a suppressor tone that was 25 Hz below the primary tone frequency and had a 10 dB higher level than that of the primary tone. Two 4.8-s-long stimuli were used, one containing the primary and suppressor tones to obtain, first, the ear-canal sound pressure due to the primary tone alone, and the other containing the primary tone only (resulting in an additional pressure component due to the SFOAE). The vector difference between these two conditions was taken as the SFOAE. The measurement of SFOAE iso-suppression curves was analogous to that of the DPOAE iso-suppression curves (also with sequentially increasing primary levels, which were 30, 35, and 40 dB SPL). First, however, two suitable primary frequencies were selected with the same selection criteria as in the “DPOAE suppression” section.

Pilot experiments revealed that 9-dB suppression of SFOAE with primary levels > 40 dB SPL often required BT levels that modulated the sensitivity of the ER-10C probe microphone (> 105 dB SPL), and those modulations (observed in the spectra of recordings made with the ER-10C probe microphone in the B&K 4157 ear simulator’s cavity) could not be distinguished from the SFOAE modulation in the recorded signal from the ear canal. Hence, BT levels required for periodic 9-dB SFOAE suppression could only be determined with primary levels up to 40 dB SPL. Analysis of the recording made with the more linear ear-simulator microphone revealed that modulation of the receivers by the BT allowed somewhat higher BT levels.

During the SFOAE biasing recording of a 20-s stimulus duration, the 55-Hz BT was switched off for the first 2 × 4.8 s to obtain the unbiased SFOAE level. The primary and suppressor tones were on during the first 4.8 s, while during the following 4.8 s, the suppressor tone was switched off. The unbiased SFOAE level served as the reference level to quantify the depth of the minimum in the SFOAE modulation pattern recorded during the remaining 10.4 s, during which the 55-Hz BT was switched on. Like the DPOAE-suppression period pattern, the SFOAE-suppression period pattern was derived by the inverse DFT of the two closest pairs of modulation sidelines and the SFOAE “carrier” in the average spectrum of the 400-ms snippets while the BT was on. The SFOAE carrier was obtained by reducing the complex-valued spectral component at the SFOAE frequency by the complex-valued sound pressure in the ear canal due to the primary tone alone (measured in the first 4.8 s of the recording when the SFOAE was suppressed by the suppressor tone).

Manual adjustment of the BT level to achieve a 9-dB SFOAE suppression depth was identical to that in the DPOAE experiment.

### Loudness Suppression

**Fig. 1:**
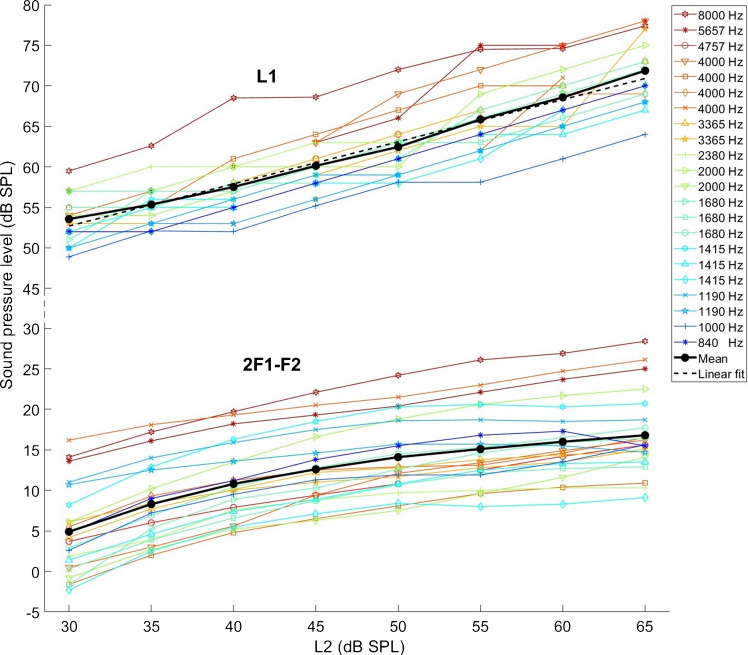
Levels of L1 that maximized the 2F1–F2 DPOAE level as L2 increased are shown in the upper set of graphs. A linear fit to the mean (L1 = 0.52*L2 + 37.1 dB) is shown as a black dashed line. The resulting 2F1–F2 levels are shown in the lower set of graphs. The average across individual ears is shown in black. For both sets of curves, the individual lines’ colors go from cold to warm with increasing frequency (see image legend). Note that each ear was measured with two F2 frequencies and has its own individual marker

**Fig. 2:**
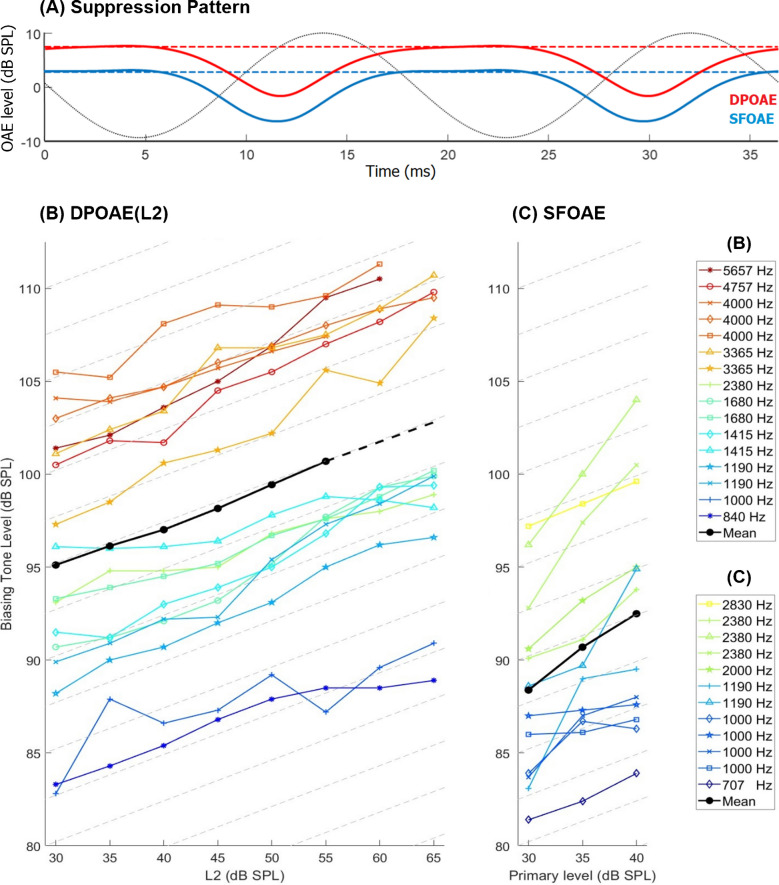
**A** Examples of periodic 9-dB suppression patterns obtained for DPOAE (red) and SFOAE (blue) in the same subject at the same primary frequency (2380 Hz, L2 and the SFOAE primary level were 40 dB SPL, BT levels were 95/94 dB SPL for DPOAE/SFOAE; subject was A2_R in Table 1). The unsuppressed OAE level (no BT, dashed lines) served as the reference for the 9-dB suppression criterion. Two periods are shown. The gray sinusoid is a BT recorded with the probe microphone in the ear canal to show its phase (plotted on an arbitrary linear scale, upward is an increase in pressure). The panels below show the required BT levels to achieve 9-dB suppression of the DPOAE (**B**) and the SFOAE (**C**) as a function of primary level. Individual ears (thin lines; *N* = 8 for DPOAE and *N* = 6 for SFOAE) have the same marker as in Fig. 1. The mean curves (solid black) were obtained only for conditions where data were available from all individuals, while their dotted extension in panel B has the average slope of cases that presented data for L2 > 55 dB SPL. The grid lines in the two lower panels have the average slope of the DPOAE (L2) shown in panel B (0.22 dB/dB)

In this psychoacoustic experiment, the level of a BT required to produce a periodic loudness suppression within a train of tone pips (with frequencies of 1 or 2 kHz; 8-Hz repetition rate) was measured. The tone pips were three cycles long, including a one-cycle long rising and a one-cycle long falling cosine ramp. Such short probe stimuli are required to resolve the phase-specific effect of the suppressor tone (See Fig. 2A). As the 55-Hz BT had a frequency that was 1-Hz less than an integer multiple of the repetition rate (i.e., 8 × *N* − 1, with *N* = 7), eight subsequent tone pips coincided with eight equal-distant phase locations within the BT cycle (see Fig. 1 in the study by Jurado et al. [20]). With sufficient BT level, this led to a perceivable 1-Hz modulation of the pip train. From a certain BT level upwards, the sensation of one of the tone pips was completely suppressed and the pip train became periodically interrupted. This gave rise to a “galloping” sensation, which was an easily recognizable criterion and thus became the target criterion of the task. When the criterion was perceived, the subject had to press a button labeled “yes” (otherwise, “no”), in a yes/no paradigm (one-interval, two-alternative forced-choice task, 1I-2AFC). Subjects had the chance to familiarize themselves with the galloping sound by listening to a pip train that was fully sinusoidally amplitude modulated at 1-Hz, in the absence of the BT. This produced a similar sensation to that of a pip train in the presence of the BT at criterion level.

The BT level was adaptively adjusted by a maximum-likelihood-tracking (MLT) procedure; each track consisted of sixteen stimulus presentations. The final psychometric function, which was derived from the sixteen Yes/No responses, was based on a cumulative Gaussian distribution, whose slope can be expressed as a standard deviation (SD). Subjects who, after ten training tracks, still consistently produced shallow psychometric functions (with SD > 3 dB) were excluded from further testing. After training, five MLT tracks were obtained per pip-train level (15–35 dB SL, 5-dB steps), and the criterion BT levels were obtained from their average. To be able to define sensation levels, individual sensation thresholds for the 1- and 2-kHz pip trains had to be measured before the biasing experiments began. For these measurements, a 2I-2AFC task with a 3-down 1-up adaptive staircase procedure was repeated four times, and the median of the obtained thresholds was taken. The stimulus paradigm and MLT procedure were described in detail in the study by Jurado et al. [20].

## Results

### Optimal L1 as a Function of L2 (No Biasing)

The average L1 levels that maximized the 2F1–F2 DPOAE increased linearly with L2 (Fig. 1). The optimal increase of L1 relative to L2 was on average 0.52 dB/dB and had a mean intercept at 37.1 dB. These values are in line with the “scissor paradigm” proposed by Kummer et al. [24]. We further noted that the intercept tended to increase with increasing primary tone frequency (*R*^2^ = 0.56, *p* < 0.001). This dependency (in line with other studies that have found frequency dependencies in optimal primary parameters, e.g., [25–28]) was quantified as ~ 3 dB per octave of F2 increase:

1 $$L1=0.52\times L2+2.93\times\log_2\left(F2\right)+4.72\left(dB\right)$$

Note that these data were obtained with a constant primary-frequency ratio of F2/F1 = 1.2. It is uncertain whether this frequency dependence in the optimum L1 would still be found when the optimal frequency ratio (which tends to decrease with frequency; [29, 38]) is simultaneously sought.

The growth of the 2F1–F2 distortion component shows the usual compressive characteristic (Fig. 1, lower set of graphs). Its slope at moderate primary levels (L2, 45–60 dB SPL) was ~ 0.2 dB/dB, in line with previous studies (e.g., [24, 31]).

### OAE Biasing

The SFOAE suppression patterns in response to the 55-Hz BT looked very similar to those of the 2F1–F2 DPOAE component (Fig. 2A). On average, the required BT levels to achieve criterion DPOAE suppression rose linearly as the L2 level increased (Fig. 2B, solid black line; *R*^2^ of a linear fit to the mean, 0.995). The average iso-suppression slope was 0.22 dB/dB (see Table 1 for individual values). This was calculated only up to 55 dB SPL where all subjects presented data; however, the slope remained practically unchanged for the available data between 55 and 65 dB SPL (0.21 dB/dB; dashed line).

**Table 1:** Individual and average slopes obtained for DPOAE(L2) and SFOAE iso-suppression. The slope values were obtained from linear fits to individual data. Asterisks indicate that the primary-level range was restricted (as shown in Fig. 4)

Subject ID	DPOAE		SFOAE	
	*F2/F1 (Hz)*	Slope (dB/dB)	*Primary frequency (Hz)*	Slope (dB/dB)
A1_L (⚝)	1190/990	0.25	1000	0.06
	3365/2760	0.30	2000	0.44
A2_R (+)	1000/835	0.16	1190	0.64
	2380/1985	0.16	2380	0.37
A2_L (o)	1680/1375	0.29	–-	
	4757/3900	0.27	–-	
A3_R (x)	1190/975	0.30	1000	0.43
	4000/3335*	0.15	2380	0.77
A4_R (*)	840/700	0.17	–-	
	5657/4635*	0.32	–-	
A5_R(◇)	1415/1160	0.26	707	0.25
	4000/3335	0.19	1000	0.24
A6_R (△)	1415/1180	0.09	1190	0.63
	3365/2805	0.27	2380	0.78
A7_L(□)	1680/1375	0.20	1000	0.08
	4000/3335*	0.19	2830	0.24
**Mean**		**0.22**		**0.41**
**SD**		**0.07**		**0.25**

Due to the exponential increase in BM compliance from base to apex, higher BT levels are required to transversally displace the BM at the stiffer, more basal locations where the TWs of higher-frequency probes behave nonlinearly. In line with this, the required BT levels for DPOAE iso-suppression markedly increased the higher the F2. In the range L2 = 30–55 dB, for which the data set is complete, a linear fit revealed a slope of 7.34 dB/octave (*R*^2^ = 0.89, *p* = 4.0 × 10^−8^), which is close to the 8.4 dB/octave obtained by Jurado et al. [20] with 15- and 30-Hz BTs.

Iso-suppression curves for SFOAEs (Fig. 2C) were obtained for six of the eight ears that also completed the DPOAE suppression measurements. As observed for the DPOAE, the average BT level increase was also practically linear for the SFOAE (*R*^2^ of linear fit to the mean, 0.995) but had a mean slope of 0.41 dB/dB (note, however, that it could only be obtained for the limited primary level range from 30 to 40 dB SPL). The latter slope is close to twice the average slope observed for DPOAE(L2) (which was 0.21 dB/dB when only the six ears for which SFOAE iso-suppression curves could also be obtained were considered, and 0.20 dB/dB when of these DPOAE data, only the L2 range for which SFOAE iso-suppression curves were measured was taken into account) and the difference was statistically significant (pairwise *t*-test on the subset with data in both measures: *T*_11_ = − 2.55, *p* = 0.027). Virtually identical to the DPOAE data, the required BT level for SFOAE iso-suppression increased with increasing primary frequency with a slope of 7.7 dB/octave (*R*^2^ = 0.86, *p* = 1.3 × 10^−5^).

No significant correlation was found across subjects between individual DPOAE and SFOAE iso-suppression slopes (Table 1; *R*^2^ = 0.06, *p* = 0.46). There was also no significant correlation between individual iso-suppression slopes obtained for the higher and the lower primary frequencies across subjects, neither for DPOAE (*R*^2^ = 0.05, *p* = 0.58) nor SFOAE (*R*^2^ = 0.30, *p* = 0.26).

### Loudness Suppression

Like the iso-suppression curves for OAE, the BT level required to just create a galloping sensation of the tone-pip train also rose linearly with increasing tone-pip level (Fig. 4A, R^2^ of a linear fit to the overall mean, 0.998). The average slope of 0.47 dB/dB (essentially the same for the lower and upper probe frequencies; see Table 2 for individual values) was similar to that of the average iso-suppression curves for SFOAE. The required BT level increased on average by 8.9 dB as the tone-pip frequency increased from 1 to 2 kHz. This is roughly in line with the dependence on primary frequency reported above for both types of OAE.

**Table 2:** Individual and average slopes obtained for loudness iso-suppression. Values correspond to linear fits across the probe-level range (15–35 dB SL)

Subject ID	Slope (dB/dB)	
	1 kHz	2 kHz
B01 (o)	0.50	0.39
B02 (□)	0.50	0.35
B03 (*)	0.52	0.71
B04 (△)	0.34	0.42
**Mean**	**0.46**	**0.47**
**SD**	**0.08**	**0.16**

## Discussion

**Fig. 3:**
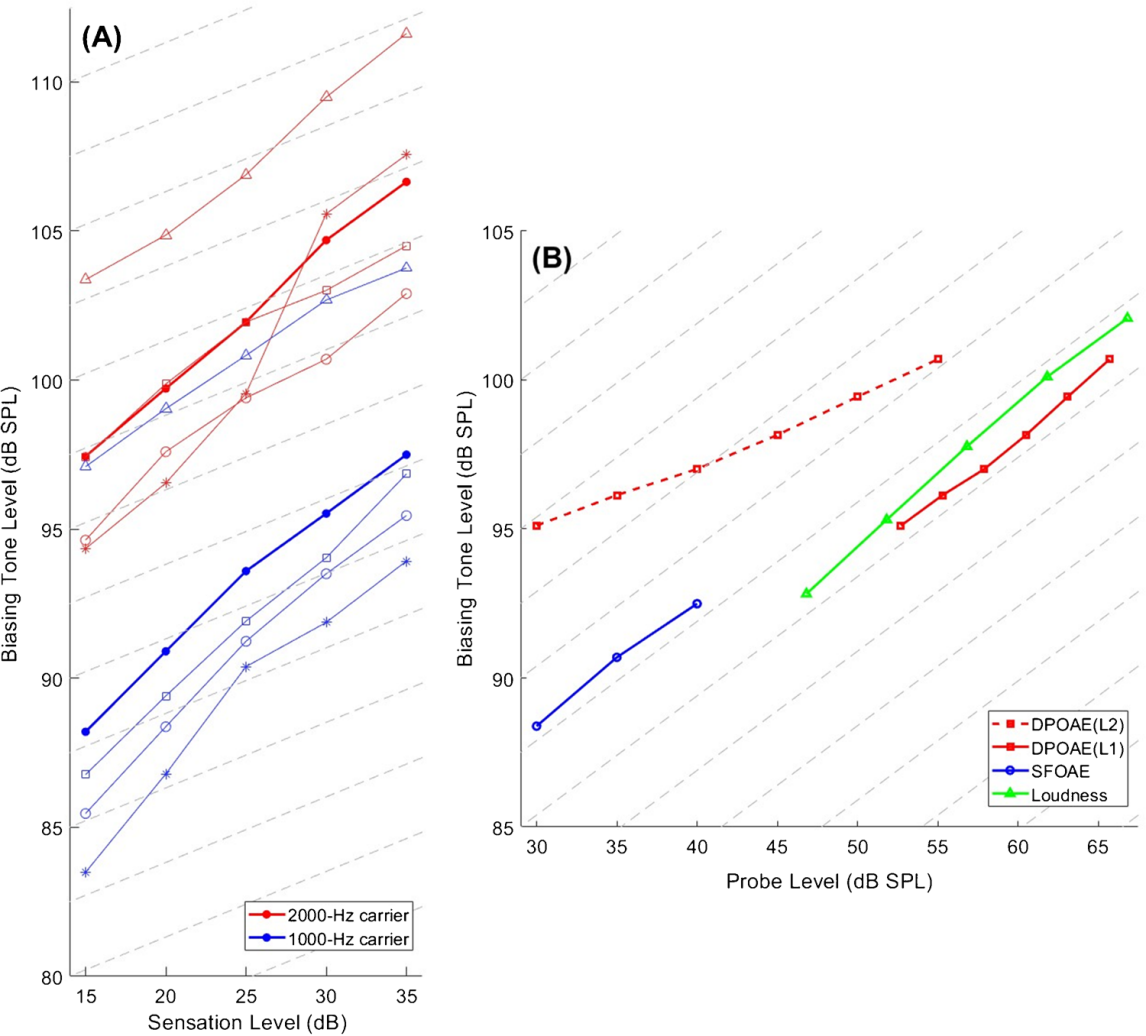
**A** Required biasing tone levels to achieve criterion suppression in the subjective loudness of pip trains as a function of pip-train sensation level (dB SL) for 1-kHz (blue) and 2-kHz (red) tone pips. Individual markers correspond to those in Table 2. (Note that this is a different subject group from that of the OAE experiments). Bold lines show mean data. The slope of the grid is the same as in Fig. 2 (0.22 dB/dB). **B** The comparison of average iso-suppression curves shows good alignment across all three experiments when DPOAE data are plotted as a function of L1. Loudness iso-suppression data for both carrier frequencies were averaged, and probe levels were converted to dB SPL by adding the average of the mean thresholds for the 1-kHz (30.3 dB pSPL) and 2-kHz (33.3 dB pSPL) pip trains to the sensation levels. Grid lines have a slope of 0.4 dB/dB

Our results reveal that the SFOAE and loudness iso-suppression curves have a very similar slope and contrast with the distinctly shallower slope of the DPOAE(L2) iso-suppression curve. Because L1 has to grow 0.5 dB per 1 dB of L2 increase (Fig. 1) to achieve maximum 2F1–F2 level, the iso-suppression curve for DPOAE compares better with the other two when converted so that L1 becomes the varied parameter (utilizing the linear fit to the mean data shown in Fig. 1; L1 = 0.52*L2 + 37.1 dB). The resulting DPOAE(L1) iso-suppression curve presents a noteworthy alignment with the loudness and SFOAE iso-suppression curves (Fig. 3B). The slope of DPOAE(L1) was 0.43 dB/dB when all subjects were included, and 0.40 dB/dB for the subset of subjects with SFOAE data. (The latter slope was not statistically different from the corresponding SFOAE iso-suppression slopes; mean, 0.41 dB/dB, *t*-test: *T*_*11*_ = − 0.079, *p* = 0.94). The better alignment might indicate that the F1-tone is of primary interest when studying DPOAE suppression. This is plausible because the 2F1–F2 emission is predominantly generated in the BM region surrounding the peak of the F2 TW [32], and this peak lies within the active region of the F1-TW (given F2/F1 = 1.2). One could conceive that this DPOAE component is produced by the F2-tone interfering with the amplification of the F1-tone, which would get modulated at their beat frequency and so, intermodulation products at 2F1–F2 and F2 are produced.

Although it is per se not clear how the 9-dB criterion for OAE suppression relates to the galloping criterion for loudness suppression, the similarity between the BT levels achieving criterion suppression for DPOAE(L1) and loudness shows that both criteria require displacement biases that are within 2 dB of each other (which was rather coincidental, since the choice of the 9-dB criterion was somewhat arbitrary). The same also holds for the 9-dB SFOAE suppression, assuming the SFOAE iso-suppression curve would continue to grow linearly at ~ 0.4 dB/dB.

### Comparison with Iso-Suppression Curves Obtained with Low-Side Suppressors > 200 Hz

Iso-suppression data of OAE by low-side suppressors (i.e., with a frequency below that of the probe) have been obtained abundantly in the past, and there also exist loudness suppression data. We compare our data here, however, only with iso-suppression measurements which utilized suppressor tones at least one octave below the probe so that the BM response to the suppressor can be assumed to grow linearly at the location of the peak and active region of the probe’s TW (like the BM response to a BT; see the “Iso-suppression curves and the non-linear growth of basilar membrane vibrations” section for more details). The difference between biasing experiments and these low-side suppression experiments is that the suppressor cycle in the latter is too short to either lead to phasic suppression or to resolve it, so that the average suppression over a suppressor cycle is analyzed.

Keefe et al. [33] obtained SFOAE suppression tuning curves (STCs) for a wide range of primary levels and also reported iso-suppression curves (3-dB suppression depth) as a function of primary level (30–60 dB SPL) with off-frequency suppressor tones that were an octave below the primary tones. They were linear, like ours, and had a slope of 0.4 dB/dB with a 1-kHz primary tone and 0.28 dB/dB with a 2-kHz primary tone, which we did not observe in our SFOAE biasing data.

Gorga et al. [34] obtained DPOAE(L2) iso-suppression curves with slightly steeper slopes of 0.26 dB/dB (F2 = 4 kHz) than those observed in our study (0.22 dB/dB), but still clearly shallower than the average slope of the SFOAE iso-suppression data reported by Keefe et al. [33]. Later, Gorga et al. [35] reported even steeper DPOAE(L2) iso-suppression slopes of 0.41 dB/dB for F2 = 500 Hz and 0.31 dB/dB for F2 = 4 kHz. The study by Gorga et al. [36] extended the range of tested probe frequencies and reported slopes of 0.2 dB/dB at 8 kHz. The reason why this dependence on probe frequencies is absent in our data is unclear. The optimization of L1 for every L2 and F2 combination could have been an explanation, but this was also done by Gorga et al. [35, 36].

Related to our loudness iso-suppression data is the classic study by Wegel and Lane (1924) [37], who observed that for tones > 2 kHz to remain audible during masking, their levels have to be increased by 2.4 dB per 1-dB increase in the level of a 400-Hz masker tone. In other words, a masker level increase of 1/2.4 = 0.42 dB is required to make a 1-dB increase in the probe level just inaudible again. This value is comparable to the slope of our average loudness iso-suppression curve (0.47 dB/dB).

Dewey and colleagues [5] measured two-tone suppression curves for the mechanical responses of BM and RL, which had almost identical tuning for near CF probe tones. Suppressor tones more than one octave below CF needed to be increased by ~ 0.5 dB per 1-dB probe level increase to maintain the probe-suppression criterion of 1.5 dB. This value is similar to the slopes of the SFOAE and loudness iso-suppression reported here and indicates that both are likely rooted in the suppression of mechanical amplification of the probe’s TW.

In summary, the BT level required to maintain equal maximum probe suppression within the suppressor cycle as the probe level increases is not substantially different from those reported for suppressor tones above 100 Hz for equal average suppression, as long as their frequencies were at least an octave below the probe frequency.

### Iso-Suppression as a Function of Probe Frequency

It must be noted that the 8.9 dB/octave slope in probe-frequency dependence that we found for loudness iso-suppression disagrees with our previous findings of just ~ 3 dB/octave obtained with the same loudness iso-suppression technique [20]. Although our in situ calibration may cause inaccurate absolute levels at the eardrum above ~ 2 kHz, this cannot explain the striking difference between these two studies, which used identical in-ear probes and in situ calibration. We hypothesized in our previous paper that the shallow slope of loudness iso-suppression is seen because very low-frequency BT suppression might be caused by OHC-generated electrical potentials, which decay with a lower spatial gradient toward the cochlear base. Note, however, that the BTs in [20] were lower than 40 Hz, so that the BM moved in-phase along its entire length and thus likely created larger potentials. Since the intracochlear potential saturates at high stimulus levels [38], we cannot be sure that BTs lower than 55 Hz would produce the same loudness iso-suppression slopes as a function of probe level as found in our present study.

The relatively steep primary-frequency dependence of both OAE iso-suppression curves (of ~ 7–8 dB/octave on average) is in line with that previously observed for DPOAE iso-suppression (8.4 dB/octave, for BTs < 40 Hz; [20]) and is thought to reflect the spatial slope of the BM excitation pattern caused by the BT [20]. The fact that with a 55-Hz BT the probe-frequency dependence of loudness iso-suppression was similar lets us conclude that the loudness suppression in this study is caused by mechanical effects on the OHC MET. It is unlikely that biasing of the velocity-sensitive inner hair cell MET by the 55-Hz BT played a role in the observed loudness suppression.

### Iso-Suppression Curves and the Non-Linear Growth of Basilar Membrane Vibrations

Researchers have commonly explained the shallow slope of OAE iso-suppression curves as a function of probe level by the compressive growth of the BM response to the probe that contrasts with the linear growth of the response to the suppressor tone at the location of the probe’s characteristic place. So, BM compression estimates were made from these data (e.g., [33, 34]). Also, the mentioned psychoacoustical finding by Wegel and Lane [37] has been interpreted in this way [39]. Of course, the suppressive interaction between tones most likely takes place at the MET of the OHC stereocilia, located at the RL, for which motion data have become recently available with OCT technology (for a review, see Olson et al. [40]). Thus, making a link between suppression data and compressive BM growth is somewhat of a simplification. However, the interpretation of the OCT-based data from within the organ of Coti is still in flux [40] because structures of the organ of Corti, including the RL, apparently move in all three spatial dimensions, but phase-sensitive OCT technology records the motion projected onto the optical axis of the beam. So, recently available semi-transverse RL data are likely poor estimates of the stimulus to the METs, which are activated by shear motion between the RL and the TM [5]. Thus, despite available RL data, some researchers (e.g., [41]) still prefer BM data when conveying basic concepts of cochlear function, as their interpretation of being largely transversal has not been challenged. We will also use the classic simplification of considering BM displacement to relate our suppression data to cochlear mechanics, although it has been shown that RL suppression of characteristic frequency (CF) tones is similarly tuned to the BM [5].

**Fig. 4:**
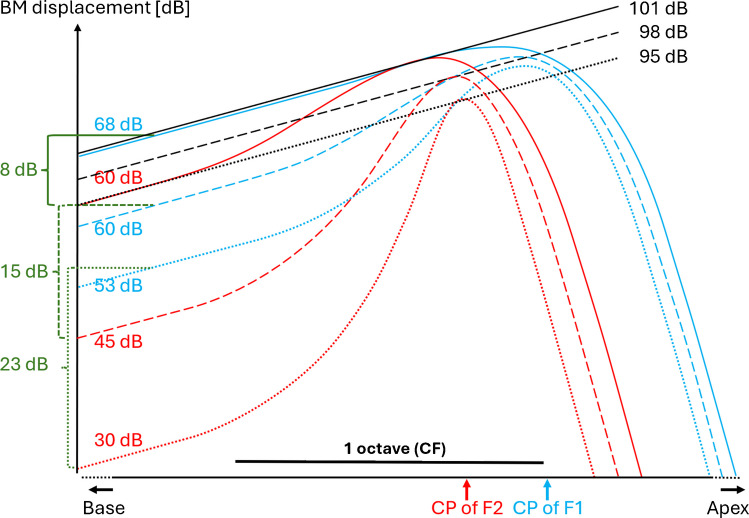
Schematic showing three triplets of TW envelopes that have equal displacement at the location of the TW peak in response to the F2 tone (red) along a section of the BM. The TWs of the F1 primary tone are shown in blue. The black lines show the BM displacement bias magnitude in response to the BT. The triplets are illustrated for L2 levels of 30 dB SPL (dotted line), 45 dB SPL (dashed line), and 60 dB SPL (solid line). Values in green show the primary level differences that on average produced the maximum 2F1–F2 emission (Fig. 1). Values in blue give the corresponding L1 levels (in dB SPL). Values in black are the BT levels (in dB SPL) that on average produced a 9-dB suppression of the 2F1–F2 emission (Fig. 2B). Locations labeled “CP” are the characteristic places where the BM is most sensitive to either the F1 or F2 primary tone. Note that the upper end points of the green brackets touch the F1–TWs accordingly more apically

Figure 4 illustrates schematically the TW envelopes for the tones involved in our DPOAE biasing experiment. The envelopes are shown only in the longitudinal section from just basal to the active region of the F2 TW to where the F1 TW has decayed by more than 40 dB (i.e., a range of ~ 2 octaves of CF). They are drawn by assuming (a) linear growth in the tail, (b) compressive growth near their peak determined from our data in Figs. 1 and 2B, and (c) identical F1 and F2 responses at the location of the F2–TW peak. Thus, on the left (basal) side of the illustration, the BM vibrations grow for all tones linearly with stimulus level, so that the sound pressure levels (taken from our results shown in Figs. 1 and 2B) are scaled correctly on the vertical BM displacement axes in decibels. The blue curves illustrate the TW envelopes in response to the F1-tone and those in red in response to the F2-tone. The growth of these TWs with stimulus level becomes increasingly compressed as they progress to their characteristic place, culminating here in a rate of 0.2 dB per dB of stimulus level increase, a value taken from the slope of our DPOAE(L2) iso-suppression curve (Fig. 2B).

Based on the widely accepted assumption that the 2F1–F2 emission reaches its maximum level when the primary tones produce equal displacement at its generation site, so that the amplitude modulation due to beating of the primary responses is 100% (reviewed [42]), the F1–TW shapes (blue) were drawn to fulfill this condition at the F2–TW peak, the approximate center of the DPOAE generation region. This is, of course, a simplification, because the DPOAE is not generated exactly at the F2–TW peak but in the surrounding it, and, in reality, the condition of equal TW amplitudes is likely a weighted compromise within this region. In accordance with the growth of the optimum L1 (Fig. 1), their amplitudes increase at half the rate of the F2–TW amplitude in the linear region (left-hand side). Motivated by the slope of our DPOAE(L1) iso-suppression curve, the growth of F1–TW amplitude at the location of the F2–TW peak shows a compression rate of 0.4 dB/dB. Measurements in gerbil cochleae agree with such spatial build-up of compression, showing that compression has not yet reached its maximum at the location where a tone of 1.2 × higher frequency would peak but is roughly just half of that at the TW peak (Fig. 6 in [41]; although with lower overall compression). Note that the TW peaks shift basally with an increasing tone level (e.g., [5, 43], Fig. 2), and we assume here that the region of DPOAE generation.

The schematic illustrates that the difference in growth rates of L1 and L2 required to maximize the 2F1–F2 DPOAE might be, in the first approximation, a compensation for the differing compression rates of the F1- and F2–TWs in the DPOAE-generation region surrounding the F2–TW peak. The absolute compression rates of the F1- and F2–TW growths in the F2–TW peak regions might be revealed by a low-frequency suppressor tone that produces a linearly growing TW amplitude within the DPOAE-generation region. The linearly growing magnitudes of the three suppressor displacements that caused 9-dB DPOAE suppression for the three primary level pairs (from Fig. 2B mean data) are shown as black lines. Although their amplitudes at the F2–TW peak are shown here equal to those of the primary-tone TWs, we actually assume only that the 9-dB DPOAE suppression is maintained when the suppressor amplitude is kept proportional to the amplitudes of the primary TWs at the F2–TW peak. The vertical spacing of the suppressor TWs shows that the suppressor level needs to rise by just 6 dB for a 30-dB increase in L2 for this condition to hold. This corresponds to a 0.2 dB/dB compression of the F2–TW growth at its peak, while an L1 increase of 15 dB corresponds to a ~ 0.4 dB/dB compression of F1–TW growth at the same location. Comparable compression rates have been derived from BM measurements in sensitive, pristine cochlear preparations in response to a tone at the CF of the measurement location and to a tone with a frequency a factor of 1.2 below the CF [44].

The similar slopes of SFOAE and loudness iso-suppression to the DPOAE(L1) iso-suppression slope indicate that both SFOAE suppression and loudness suppression are also taking place in the active region of the probe-tone TW, where the suppressor tone likely hampers its amplification. Recent experiments by Goodman and colleagues [45] confirm that the SFOAE is largely generated in the active region of the primary’s TW. Evidence for a SFOAE generation site slightly basal to the primary’s characteristic place also comes from suppression tuning curves (STC) obtained by Keefe and colleagues [33], showing that the lowest suppression thresholds were obtained with suppressor tones slightly higher than the probe’s frequency. BM and RL responses to a CF tone are also most efficiently reduced by a suppressor tone that has its characteristic place in the active region of the CF tone [5]. Not surprisingly, the same is found in the auditory nerve [46]. In summary, only the DPOAE(L2) iso-suppression slope (~ 0.2 dB/dB) can be thought to reflect the compression rate at the TW peak because the F2–TW peak is located in the active region of the F1 tone.

The association between the compression rate in the growth of the BM response in the active region and the slopes of both the loudness iso-suppression curves and the OAE iso-suppression curves assumes that equal suppression occurs when suppressor vibration grows proportionally to probe vibration in the active region of the probe’s TW. The physiological reason why this should be the case is not obvious to us. Furthermore, the compression rate of BM growth typically increases with stimulus level, whereas iso-suppression slopes (also those published by others) are fairly invariant with probe level. Thus, confirmation by simultaneous measurements of OAE suppression and direct BM displacement in an animal cochlea is still required to confirm that the slopes of these iso-suppression curves indeed reveal rates of cochlear compression.

## Data Availability

Collected data from all experiments, as well as the code used to collect it, are available upon request.

## Declarations

### Declaration regarding the use of AI

No AI tools were used.
